# Intelligent Construction Technology Adoption Driving Strategy in China: A Tripartite Evolutionary Game Analysis

**DOI:** 10.1155/2022/9372443

**Published:** 2022-10-11

**Authors:** Jiawei Zhang, Lihong Li

**Affiliations:** College of Management, Shenyang Jianzhu University, Shenyang 110168, China

## Abstract

The adoption of intelligent construction technology (ICT) is regarded as one of the important strategies for the transformation and upgrading of the Chinese construction industry and the achievement of high-quality development. In the ICT adoption process, the government is the driving subject, the owner is an important subject, and ICT is applied in practice by the general contractor. This study first analyses the evolutionary process and the impact of participants' strategy choices on the system equilibrium by establishing a tripartite evolutionary game framework which includes the government, the owner, and the general contractor as the main stakeholders; then tests the feasibility and rationality of the model by analysing the ESS corresponding to the three phases of ICT adoption. The results show that the conditions for each ESS to be established mainly depend on the relationship between the costs and benefits of each stakeholder, and that owners are more sensitive to government subsidies and penalties than general contractors, so the government should establish a dynamic reward and punishment mechanism based on the results of the model. High adoption costs are a key barrier to ICT adoption for both owners and general contractors. This paper provides a new framework for research related to ICT adoption and a reference for the strategic adjustment of stakeholders in ICT adoption.

## 1. Introduction

Construction, as a pillar industry in China, still suffers from the low level of information technology, sloppy management methods, high energy consumption, and high pollution [1]. The traditional building construction methods will lead to large amounts of energy consumption, waste, and greenhouse gas emissions, which aggravate the global greenhouse effect [2]. According to McKinsey & Company's 2016 industry report [3], the construction industry is only more computerized than agriculture, investing less than 1% of its total revenue in research, innovation, and new technologies. It is still considered that the construction industry is in the Industry 1.0 era, as new digital technologies have not been adopted overall [4]. Bouck reports [5] that the construction industry demands the integration of intelligent devices more than other industries and the information fragmentation and collaboration among its participants are the highest. This requires the construction industry to achieve digitalization as soon as possible, applying new technologies to communicate more effectively between stakeholders and thus improve efficiency. With the new round of technological revolution, the new generation of information technology represented by artificial intelligence, big data, the Internet of Things, blockchain, and intelligent machines is accelerating its penetration into the construction industry [6], and deep digital transformation and upgrading has become the key to achieving high-quality development in China's construction industry. It can be seen that the development of intelligence in the construction industry is inevitable, and this is confirmed by the emergence of a large number of ICTs (intelligent construction technologies).

In essence, the adoption of ICT is a huge and complex system project, a complex game process involving the formation and distribution of the interests of multiple parties, and its development depends on the balanced distribution of interests under the integration of multiple objectives and the cooperation driven by the collaboration of the main parties. As the policy maker and promoter of intelligent construction, the government plays the role of incentive, support, and supervision in promoting the adoption of ICT, and its goal is to maximize social benefits; for owners and contractors, the adoption of ICT depends on their input costs and external benefits. There are many different contracting models in the construction industry and in this study, we consider the behavioural strategies used by stakeholders to promote ICT adoption in the general contracting model for engineering projects. Therefore, this study considers that the government is the main body promoting the adoption of ICT, the owner is the important body to adopt ICT, and the general contractor is the main body for the implementation of the practical application of ICT. The different goals and interests of stakeholders in the adoption of ICT, as well as the influence of limited rationality, make it necessary to study the behavioural strategies made by governments, owners, and general contractors in the dynamic evolutionary environment to achieve the sustainable development of ICT.

At present, there are few studies on the strategies of stakeholders in promoting the adoption of ICT. To further improve the research in this area, this study examines the interaction patterns and decision-making behaviour of three stakeholders—the government, owners, and general contractors—in different contexts based on the theory of the tripartite evolution game. We are dedicated to addressing the following three questions: (i) what is the payoff matrix in the tripartite evolution game model considering the benefits and costs of the government, owners, and general contractors? (ii) What are the evolutionary stable strategies (ESS) and the corresponding conditions for achieving the adoption of ICT? (iii) What are the effects of different parameter changes on the behaviour and decisions of the evolutionary game players? To address the mentioned questions, we built a tripartite evolutionary game model consisting of the government, owners, and general contractors and calculated the payoff matrices of the three players; then, we obtained the ESS and its establishment conditions by calculating the replication dynamic equations and the Jacobi matrix; secondly, we used numerical simulations to confirm the reliability of the evolutionary game model and further explored the effects of the model parameters on the strategic behaviour of the three players.

The rest of the paper is organized as follows: Section 2 is a review of the existing literature on this study.  Section 3 is devoted to the construction of a tripartite evolutionary game model, the analysis of the stability of each subject's strategy, and the possible ESS.  Section 4 conducts numerical simulations.  Section 5 proposes strategies to promote the adoption of ICT based on the research results.  Section 6 concludes the research, presenting the limitations in the research and future directions for development.

## 2. Literature Review

It can be roughly divided into three categories based on the existing literature: intelligent construction technologies, stakeholders in the adoption of ICT, and evolutionary game theory. ICT is the digital transformation of elements and resources in construction, which is reflected in the efficient coordination of project planning, design, construction, operation, and maintenance [7]. Han and Wang [8] argue that the core step of intelligent construction is to visualize the construction project by collecting data to assist the relevant parties in decision-making. Deng et al. [9] propose blockchain technology as one of the fundamental components of intelligent transformation in the construction industry, and it has the potential to address issues related to procurement and supply chain, big data, and data storage [10]. As can be seen, intelligent construction is the combination of advanced technologies such as BIM, IoT, blockchain, and 4D visualisation technology with each other to achieve integrated project management, which promotes collaboration between relevant parties and the efficient use of resources and energy throughout the construction process and promotes the sustainable development of the entire construction industry.

### 2.1. Research on Technologies Related to Intelligent Construction

In response to the definition of intelligent construction, many technologies have been developed to improve construction efficiency, optimise the configuration of construction machinery, and the overall management of projects [11]. Research on intelligent construction technologies often starts from specific application points: to promote intelligent transformation in the construction industry, Li et al. [12] introduce an extended sociotechnical framework for the implementation of blockchain in the construction industry. Han and Wang [8] introduce big data technology to the field of smart construction, exploring the difficulties and technical framework of big data applications. Kochovski and Stankovski [13] establish an edge computing architecture that can be used to support an intelligent construction environment, facilitating project information management, and communication between various parties involved. To give the machines used in building sites autonomy and awareness, Rossi et al. [14] developed smart sensor devices. To establish an IoT cloud-based platform for big civil engineering projects, Bucchiarone et al. [15] suggest using IoT technology. This would enable project managers to remotely oversee several construction sites in various places simultaneously. Zhang and Man [16] developed a BIM-based 4D progress control system for prefabricated buildings by addressing technological issues such as the connection of 3D models and progress information. Wang et al. [7] proposed an intelligent construction system for prefabricated components based on the Internet of Things (ICSPB-IoT), which offers cross-phase, cross-organizational information interaction for government supervision departments, contractors, and owners.

Through the above literature analysis, this study concludes that ICT is a modern advanced technological tool that enables human-computer interaction, data interoperability and sharing, and integrated construction management. Current ICT typically includes big data and blockchain, intelligent construction machinery for human-machine collaboration, construction IoT, and BIM technology.

### 2.2. Stakeholders in the Adoption of ICT

All stakeholders contribute in some way to driving the adoption of ICT, and stakeholders need to be aware of the benefits of implementing something new before it can be adopted.

Based on Ackermann's definition of stakeholder [17], stakeholders for ICT adoption are defined in this study as groups, organizations, and individuals who have the ability to influence, are influenced by, or have a significant interest in it. Ullah et al. [18], through a literature review, identified four key stakeholders for ICT adoption in the real estate industry as consumers, contractors and owners, government and regulatory agencies, and complementary industries. In a conceptual framework for an IoT-based intelligent construction system for prefabricated buildings (ICSPB-IoT), stakeholders are considered to include government supervision departments, owners, and contractors [7]. Through a literature review, Prebanić and Vukomanović [19] argue that the main stakeholders in the use of ICT for digital management cover governments, contractors, and owners. In mega sustainable construction projects (MSCP) applying ICT, governments and designers are considered to have a significant influence on other stakeholders and play the role of key stakeholders [20]. The owner is often seen to be able to influence contractors in the form of promoting innovation [21]. With government help, construction enterprises prefer to speed up the implementation and acceptance of digitalization technology [22]. It is important for policymakers to consider factors such as technology awareness and leadership when developing policies to integrate ICT into construction projects [23]. The adoption of new technologies by owners is driven by economics, as they are cost-sensitive and profit-oriented [24]. The key success factors for contractors to adopt new technologies and implement sustainable construction are policies established by authorities, cost control, etc [25].

Based on previous research, the government, the owner, and the general contractor are considered as key stakeholders in the ICT adoption process. In this process, the government is a very important stakeholder as it plays a decision-making and monitoring role as a policy maker. The owner, as the construction unit of the project, undertakes the task of managing the construction of the project during its implementation and it has the right to decide whether to take the initiative to adopt ICT in the construction of the project. The general contractor, as the actual contractor of the project, is cost-sensitive and may take a negative attitude towards ICT despite pressure from the government or the owner, and therefore plays a decisive role in the ICT adoption process. It is also one of the key stakeholders in the ICT adoption process.

### 2.3. Evolutionary Game Theory

To examine the effect of incentives and regulation, evolutionary game theory is a useful tool [26]. In contrast to classical game theory, evolutionary game theory assumes that all players are finitely rational, aim to maximize their benefits, and constantly adjust their strategies through imitation and learning in the game to eventually reach a dynamic equilibrium. Thus, the evolutionary game theory allows for the analysis of the strategic choices of multiple players from a dynamic perspective. It has two basic concepts: replication dynamic equations and evolutionary stable strategy (ESS) [27].

Evolutionary game theory has been used as a research tool in a wide variety of different research areas, including the diffusion of novel technologies. Currently, research on technology adoption in the construction industry has focused on green building technology (GBT), BIM adoption, and prefabricated construction, as shown in Table 1.

According to the above analysis, evolution game theory is an excellent method used to analyse mechanistic issues, and by sorting out the interests of the participating subjects, the evolutionary laws and directions of each subject can be determined. The study of the adoption of new technologies through evolutionary game theory is well established and therefore it is also applicable to this research.

Existing research is less likely to combine ICT adoption with evolutionary game theory. Firstly, the government, owner, and the general contractor do not have a clear understanding of each other's intentions, and the three stakeholders have limited information, knowledge, and resources, and they are all limited rational players. Secondly, as policies and measures to promote ICT adoption are still evolving, in the process of adopting technology, the three players will modify their tactics through trial and error, imitation, and learning in various situations, realizing a long-term dynamic game. This study analyses the evolution of the system equilibrium strategy and the influence of the main parameters on the system equilibrium and the subjects' strategy choice by constructing a tripartite evolutionary game model of the government, the owner and the general contractor.

## 3. Tripartite Evolutionary Game Model

**Figure 1:**
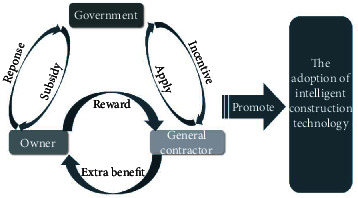
The action mechanism between the government, owner, and general contractor of ICT adoption.

The government, the owner. and the general contractor have their own different interests, which determines that there are certain conflicts and contradictions in the process of promoting ICT adoption, and the interaction between the three participants in the technology adoption process is similar to a game rather than a multilateral negotiation, because the three are not always on an equal footing [34]. The relationship between the three players is shown in Figure 1. In this section, firstly, based on the analysis of stakeholders in Section 2, we propose the assumptions of the evolutionary game model involving the government, the owner, and the general contractor and relevant parameters; then we establish the payoff matrix to calculate the replication dynamic equations of each subject and calculate the equilibrium point; finally, we obtain the possible evolutionary stable strategies (ESS) and judge the stability of the ESS through the Jacobi matrix.

### 3.1. Model Assumptions

Based on the analysis of ICT adoption stakeholders in Section 2, the following assumptions are proposed.

**Assumption 1 .**

The three parties involved in the adoption of ICT include government, owner, and the general contractor. All three parties are finite rational and can independently adopt behavioural strategies and change them dynamically. The only criterion for all players to make decisions is to maximize their interests, and all parties in the game dynamically adjust their strategies by learning, imitating, and communicating with each other at different stages of ICT development.

**Assumption 2 .**

It is assumed that the government has two strategies: active promotion and passive promotion. Active promotion means that the government pays extra for the promotion of ICT, including special funding, land concessions, tax incentives for owners that adopt ICT, and some financial compensation for general contractors who use ICT. Passive promotion means that the government does not provide substantial incentives due to financial constraints and cost pressures, but only provides publicity and encouragement.

**Assumption 3 .**

Owners can adopt ICT proactively or passively. Owners may adopt ICT proactively due to financial subsidies from the government, brand image enhancement, etc. Otherwise, they may choose to adopt ICT passively because of the high development costs and investment risks associated with the adoption of new technologies.

**Assumption 4 .**

The two pure strategies for general contractors are effort and lack of effort towards ICT application The reasons why general contractors choose to apply ICT may include their recognition of ICT or out of the pressure exerted by the owner, who, in the context of government subsidies, may offer a degree of rewards to general contractors who make an effort to apply ICT or impose high penalties for those who do not. Otherwise, they may opt for traditional construction methods.

**Assumption 5 .**

The probability of “active promotion” is *x*(0 ≤ *x* ≤ 1) and the probability of “passive promotion” is 1 − *x*; the probability of “active adoption” is *y*(0 ≤ *y* ≤ 1) and the probability of “passive adoption” is 1 − *y*; the probability of “effort” is *z*(0 ≤ *z* ≤ 1) and the probability of “lack of effort” is 1 − *z*.

### 3.2. Model Establishment and Equilibrium Solution

We set the relevant parameters of the tripartite game with the specific meanings shown in Table 2 to explore the benefits and costs under different behavioural strategies of the three stakeholders based on the above assumptions.

Based on the above analysis of three stakeholders' benefit and cost parameters, the game payoff matrix for the government, the owner and the general contractor under different strategy choices is shown in Table 3.

Based on the analysis of the payoff matrix in Table 3, we assume that the expected benefit when the government is actively promoting is *U*_11_ and the expected benefit when it is passively promoting is *U*_12_, with the average expected benefit represented by *U*_1_.

(1) $$\begin{array}{c} U_{11}=yz\left(R_{2}-C_{1}-G_{1}-G_{2}\right)+y\left(1-z\right)\left(R_{1}+P_{2}-C_{1}-G_{1}\right)+\left(1-y\right)z\left(R_{1}-C_{1}-G_{2}+P_{1}\right)+\left(1-y\right)\left(1-z\right)\left(P_{1}+P_{2}-C_{1}\right), \end{array}$$

(2) $$\begin{array}{c} U_{12}=yz\left(R_{2}\right)+y\left(1-z\right)\left(R_{1}\right)+\left(1-y\right)z\left(R_{1}-L\right)+\left(1-y\right)\left(1-z\right)\left(-L\right), \end{array}$$

(3) $$\begin{array}{c} U_{1}={xU}_{11}+\left(1-x\right)U_{12}. \end{array}$$

According to Equations (1)–(3), the replication dynamic equation for the government to promote the adoption of ICT is as follows:

(4) $$\begin{array}{c} F\left(x\right)=\frac{dx}{dt}=x\left(U_{11}-U_{1}\right)=x\left(1-x\right)\left(U_{11}-U_{12}\right)=x\left(x-1\right)\left(C_{1}-L-P_{1}-P_{2}+G_{1}y+G_{2}z+Ly+P_{1}y+P_{2}z\right). \end{array}$$

Then, assume that the expected benefit of proactive ICT adoption by owners is *U*_21_, the expected benefit of passive ICT adoption is *U*_22_, and the average expected benefit is *U*_2_.

(5) $$\begin{array}{c} U_{21}=xz\left(G_{1}+S_{2}-C_{2}-B_{1}\right)+x\left(1-z\right)\left(G_{1}+S_{2}-C_{2}-B_{2}\right)+\left(1-x\right)z\left(S_{2}-C_{2}-B_{1}-D\right)+\left(1-x\right)\left(1-z\right)\left(S_{2}-C_{2}-B_{2}-D\right), \end{array}$$

(6) $$\begin{array}{c} U_{22}=xz\left(S_{1}-P_{1}-C_{3}-B_{1}\right)+x\left(1-z\right)\left(S_{1}-P_{1}-C_{3}-B_{2}\right)+\left(1-x\right)z\left(S_{1}-C_{3}-B_{1}\right)+\left(1-x\right)\left(1-z\right)\left(S_{1}-C_{3}-B_{2}\right), \end{array}$$

(7) $$\begin{array}{c} U_{2}={yU}_{21}+\left(1-y\right)U_{22}. \end{array}$$

According to Equations (5)–(7), the replication dynamic equation for the owner's adoption of ICT is as follows:

(8) $$\begin{array}{c} F\left(y\right)=\frac{dy}{dt}=y\left(U_{21}-U_{2}\right)=y\left(1-y\right)\left(U_{21}-U_{22}\right)=y\left(1-y\right)\left(C_{3}-C_{2}-D-S_{1}+S_{2}+Dx+G_{1}x+P_{1}x\right). \end{array}$$

Finally, it is assumed that the expected benefit of the general contractor's efforts to apply ICT is *U*_31_, the expected benefit of lack of effort is *U*_32_, and the average expected benefit is *U*_3_.

(9) $$\begin{array}{c} U_{31}=xy\left(G_{2}+S_{3}+B_{1}-C_{4}\right)+x\left(1-y\right)\left(G_{2}+S_{3}+B_{1}-C_{4}\right)+\left(1-x\right)y\left(S_{3}+B_{1}-C_{4}\right)+\left(1-x\right)\left(1-y\right)\left(S_{3}+B_{1}-C_{4}\right), \end{array}$$

(10) $$\begin{array}{c} U_{32}=xy\left(S_{3}-P_{2}\right)+x\left(1-y\right)\left(S_{3}-P_{2}\right)+\left(1-x\right)y\left(S_{3}\right)+\left(1-x\right)\left(1-y\right)\left(S_{3}\right), \end{array}$$

(11) $$\begin{array}{c} U_{3}={zU}_{31}+\left(1-z\right)U_{32}. \end{array}$$

According to Equations (9)–(11), the replication dynamic equation for the application of ICT by general contractors is as follows:

(12) $$\begin{array}{c} F\left(z\right)=\frac{dz}{dt}=z\left(U_{31}-U_{3}\right)=z\left(1-z\right)\left(U_{31}-U_{32}\right)=z\left(1-z\right)\left(B_{1}-C_{4}+G_{2}x+P_{2}x\right). \end{array}$$

### 3.3. Analysis of Tripartite Stabilisation Strategies

According to the above equation and based on the stability principle of the differential equation, the stability strategy analysis is carried out for the three parties of the government, the owner, and the general contractor. The specific analysis is as follows.

#### 3.3.1. Asymptotic Stability Analysis of the Government

According to the government replication dynamic Equation (4) constructed in the previous section, the government needs to satisfy the condition of *F*(*x*) = 0, *dF*(*x*)/*dx* < 0 to achieve an evolutionary stable strategy. When  *y* = *L* + *P*_1_ + *P*_2_ − *C*_1_ − *G*_2_*Z* − *P*_2_*Z*/*G*_1_ + *P*_1_ + *L* = *y*^∗^, at this point *F*(*x*) ≡ 0, it shows that whichever strategy the government chooses belongs to the stable strategy, will get the same benefit and will not change over time, at this point all *x* are stable. If  *y* ≠ *L* + *P*_1_ + *P*_2_ − *C*_1_ − *G*_2_*Z* − *P*_2_*Z*/*G*_1_ + *P*_1_ + *L*, let *F*(*x*) = 0 to get two steady states of *x* = 0 or *x* = 1. When  *y* > *L* + *P*_1_ + *P*_2_ − *C*_1_ − *G*_2_*Z* − *P*_2_*Z*/*G*_1_ + *P*_1_ + *L*, *dF*(*x*)/*dx*_|*x* = 0_ < 0, *dF*(*x*)/*dx*_|*x* = 1_ > 0, at this point *x* = 0 is the equilibrium point, which indicates that the probability of the government choosing the active promotion ICT adoption strategy is decreasing and eventually the government will choose the passive promotion strategy. When *y* < *L* + *P*_1_ + *P*_2_ − *C*_1_ − *G*_2_*Z* − *P*_2_*Z*/*G*_1_ + *P*_1_ + *L*, *dF*(*x*)/*dx*_|*x* = 0_ > 0, *dF*(*x*)/*dx*_|*x* = 1_ < 0, at this point *x* = 1 is the equilibrium point, indicating that there is an increasing probability that the government will choose to adopt the active strategy, and eventually the government chooses the active promotion strategy.

#### 3.3.2. Asymptotic Stability Analysis of the Owner

Similarly, it can be seen that the necessary condition that owners need to satisfy in order to achieve an evolutionary stable strategy is *F*(*y*) = 0, *dF*(*y*)/*dy* < 0. When  *x* = *C*_2_ + *D* + *S*_1_ − *C*_3_ − *S*_2_/*D* + *G*_1_ + *P*_1_ = *x*^∗^, at this time *F*(*y*) ≡ 0, it shows that the owner's choice of proactive and passive adoption of ICT brings the same benefit, both are stable strategies that do not change over time, and all *y* are stable. If *x* ≠ *C*_2_ + *D* + *S*_1_ − *C*_3_ − *S*_2_/*D* + *G*_1_ + *P*_1_, let *F*(*y*) = 0, two steady states are obtained with *y* = 0 or *y* = 1. When *x* > *C*_2_ + *D* + *S*_1_ − *C*_3_ − *S*_2_/*D* + *G*_1_ + *P*_1_, *dF*(*y*)/*dy*_|*y* = 0_ > 0, *dF*(*y*)/*dy*_|*y* = 1_ < 0, at this point *y* = 1 is the equilibrium point, which indicates that the owner's strategy choice transitions from passive to proactive, eventually choosing proactive adoption. When *x* < *C*_2_ + *D* + *S*_1_ − *C*_3_ − *S*_2_/*D* + *G*_1_ + *P*_1_,  *dF*(*y*)/*dy*_|*y* = 0_ < 0, *dF*(*y*)/*dy*_|*y* = 1_ > 0, at this point *y* = 0 is the equilibrium point, indicating that owners are less and less likely to choose the proactive ICT adoption strategy and eventually tend to choose the passive adoption strategy.

#### 3.3.3. Asymptotic Stability Analysis of the General Contractor

Similarly, the necessary condition that needs to be satisfied for the general contractor to reach an evolutionary stable strategy is *F*(*z*) = 0, *dF*(*z*)/*dz* < 0. When *x* = *C*_4_ − *B*_1_/*G*_2_ + *P*_2_ = *x*^∗^, then *F*(*z*) ≡ 0, it shows that whether the general contractor chooses effort is a stable strategy, will get the same benefit and will not change with time, at this time all *z* is in stable state. If *x* ≠ *C*_4_ − *B*_1_/*G*_2_ + *P*_2_, let *F*(*z*) = 0, two steady states are obtained with *z* = 0 or *z* = 1. When *x* > *C*_4_ − *B*_1_/*G*_2_ + *P*_2_, *dF*(*z*)/*dz*_|*z* = 0_ > 0, *dF*(*z*)/*dz*_|*z* = 1_ < 0, at this point *z* = 1 is the equilibrium point, which indicates that the general contractor's choice of ICT adoption strategy gradually tends towards effort and eventually chooses effort. When *x* < *C*_4_ − *B*_1_/*G*_2_ + *P*_2_,  *dF*(*z*)/*dz*_|*z* = 0_ < 0, *dF*(*z*)/*dz*_|*z* = 1_ > 0, at this point *z* = 0 is the equilibrium point, indicating that the probability of the general contractor choosing to make an effort about ICT adoption gradually decreases and eventually decides not to make an effort.

### 3.4. System Equilibrium Solution Analysis

To further discuss the evolutionary equilibrium point, i.e. the equilibrium solution of this dynamic evolutionary game model, Equations (4), (8), and (12) are combined and made equal to 0. The simultaneous equations are as follows.

(13) $$\begin{array}{c} \left\{\begin{array}{c} F\left(x\right)=\frac{dx}{dt}=x\left(x-1\right)\left(C_{1}-L-P_{1}-P_{2}+G_{1}y+G_{2}z+Ly+P_{1}y+P_{2}z\right)=0,\\ F\left(y\right)=\frac{dy}{dt}=y\left(1-y\right)\left(C_{3}-C_{2}-D-S_{1}+S_{2}+Dx+G_{1}x+P_{1}x\right)=0,\\ F\left(z\right)=\frac{dz}{dt}=z\left(1-z\right)\left(B_{1}-C_{4}+G_{2}x+P_{2}x\right)=0. \end{array}\right. \end{array}$$

According to Equation (13), 12 possible equilibrium points of the tripartite evolutionary game between the government, the owner, and the general contractor can be obtained: S_1_(0,0,0), S_2_(1,0,0), S_3_(0,1,0), S_4_(0,0,1), S_5_(1,1,0), S_6_(1,0,1), S_7_(0,1,1), S_8_(1,1,1), S_9_((-B_1_ + C_4_)/(G_2_ + P_2_), 1, -(C_1_ + G_1_-P_2_)/(G_2_ + P_2_)), S_10_((C_2_-C_3_ + D + S_1_-S_2_)/(D + G_1_ + P_1_), (L-C_1_ + P_1_ + P_2_)/(G_1_ + L + P_1_),0), S_11_(-(B_1_-C_4_)/(G_2_ + P_2_), 0, (L-C_1_ + P_1_ + P_2_)/(G_2_ + P_2_)), S_12_((C_2_-C_3_ + D + S_1_-S_2_)/(D + G_1_ + P1), -(C_1_ + G_2_-L-P_1_)/(G_1_ + L + P_1_),1). Also, there may exist mixed solutions *S*∗(*x*∗, *y*∗, *z*∗) that conform to Equation (14), which are not conducive to analysis due to the complexity of the solution process of the mixed solutions. Therefore, only eight special pure strategy equilibrium points are used as the object of study in this study.

(14) $$\begin{array}{c} \left\{\begin{array}{c} C_{1}-L-P_{1}-P_{2}+G_{1}y+G_{2}z+Ly+P_{1}y+P_{2}z=0,\\ C_{3}-C_{2}-D-S_{1}+S_{2}+Dx+G_{1}x+P_{1}x=0,\\ B_{1}-C_{4}+G_{2}x+P_{2}x=0. \end{array}\right. \end{array}$$

Since it has not yet been determined whether the eight equilibrium points proposed above are equilibrium solutions of the system, we can verify whether it is an evolutionary stable strategy (ESS) by the partial stability of the Jacobi matrix of the system, according to the method proposed by Friedman [35]. According to the Lyapunov stability theory: (1) When all eigenvalues *λ* > 0, the equilibrium point is unstable. (2) When there are positive and negative eigenvalues *λ*, the equilibrium point is still unstable and a saddle point. (3) When all eigenvalues *λ* < 0, the equilibrium point is a stable point and ESS.

The Jacobi matrix *J*(*x*, *y*, *z*) corresponding to the tripartite evolutionary game model in this study is shown below.

(15) $$\begin{array}{c} J=\left[\begin{array}{ccc} J_{1} & J_{2} & J_{3}\\ J_{4} & J_{5} & J_{6}\\ J_{7} & J_{8} & J_{9} \end{array}\right]=\left[\begin{array}{ccc} \frac{\partial F\left(x\right)}{\partial x} & \frac{\partial F\left(x\right)}{\partial y} & \frac{\partial F\left(x\right)}{\partial z}\\ \frac{\partial F\left(y\right)}{\partial x} & \frac{\partial F\left(y\right)}{\partial y} & \frac{\partial F\left(y\right)}{\partial z}\\ \frac{\partial F\left(z\right)}{\partial x} & \frac{\partial F\left(z\right)}{\partial y} & \frac{\partial F\left(z\right)}{\partial z} \end{array}\right]=\left[\begin{array}{ccc} \left(2x-1\right)\left(C_{1}-L-P_{1}-P_{2}+G_{1}y+G_{2}z+Ly+P_{1}y+P_{2}z\right) & x\left(x-1\right)\left(G_{1}+L+P_{1}\right) & x\left(x-1\right)\left(G_{2}+P_{2}\right)\\ y\left(1-y\right)\left(D+G_{1}+P_{1}\right) & \left(1-2y\right)\left(C_{3}-C_{2}-D-S_{1}+S_{2}+Dx+G_{1}x+P_{1}x\right) & 0\\ z\left(1-z\right)\left(G_{2}+P_{2}\right) & 0 & \left(1-2z\right)\left(B_{1}-C_{4}+G_{2}x+P_{2}x\right) \end{array}\right]. \end{array}$$

By bringing each of the eight equilibrium points S_1_(0,0,0), S_2_(1,0,0), S_3_(0,1,0), S_4_(0,0,1), S_5_(1,1,0), S_6_(1,0,1), S_7_(0,1,1) and S_8_(1,1,1) into the Jacobi matrix, the eigenvalues of the corresponding matrices were obtained as shown in Table 4.

From Table 4, we can see that *C*_1_ + *G*_1_ + *G*_2_ > 0, according to the above principles of determination, the point (1,1,1) can only be a saddle point or an unstable point, not an ESS. This suggests that active promotion by the government, proactive adoption by the owner, and efforts by the general contractor are not evolutionary stabilization strategies. Under certain conditions, the other six points may become ESS, and the choice of each ESS is primarily determined by the trade-off between costs and benefits for each stakeholder, but each potential ESS in this game cannot exist simultaneously because of certain conflicting stability conditions for each point.

1. Stability analysis of (0,0,0) point

When (0,0,0) is ESS and the strategy chosen by the three parties is (passive promotion, passive adoption, lack of effort), at which point *λ*_1_, *λ*_2_, *λ*_3_ are all less than 0, we can obtain

(16) $$\begin{array}{c} \left\{\begin{array}{c} B_{1}<C_{4},\\ L+P_{1}+P_{2}<C_{1},\\ S_{2}-S_{1}<C_{2}-C_{3}+D. \end{array}\right. \end{array}$$

The three inequalities in Equation (16) indicate that: (1) when the reward for the general contractor's effort is less than the cost, they will choose a lack of effort strategy. (2) When the cost to the government of choosing to actively promote outweighs the damage caused by passivity and the penalties for passive adoption by owners and lack of effort by general contractors, the government will abandon the choice of the active promotion strategy. (3)The benefits to the owner of adopting ICT are less than the cost, so the owner will choose a passive ICT adoption strategy.

(2) Stability analysis of (1,0,0) point

When (1,0,0) is ESS and the strategy chosen by the three parties is (active promotion, passive adoption, lack of effort), at which point *λ*_1_, *λ*_2_, *λ*_3_ are all less than 0, we can obtain

(17) $$\begin{array}{c} \left\{\begin{array}{c} L>C_{1}-\left(P_{1}+P_{2}\right),\\ C_{4}>B_{1}+G_{2}+P_{2},\\ S_{1}-\left(C_{3}+P_{1}\right)>S_{2}+G_{1}-C_{2}. \end{array}\right. \end{array}$$

The three inequalities in Equation (17) indicate that: (1) when the owner and the general contractor choose passive adoption and a lack of effort strategy, the total social loss caused by the government choosing a passive strategy is greater than the total cost when the government actively promotes it. (2) The cost of the general contractor's efforts outweighs the incentives and subsidies received. (3) The left side of the inequality is the benefit of passive adoption minus the sum of excess costs and penalties, representing the net benefit to owners of choosing a passive adoption strategy, and the right side is the net benefit when adopting proactively, so the inequality implies that the net benefit to owners of choosing a passive adoption strategy is greater than the net benefit of adopting proactively.

(3) Stability analysis of (0,1,0) point

When (0,1,0) is ESS and the strategy chosen by the three parties is (passive promotion, active adoption, no effort), at which point *λ*_1_, *λ*_2_, *λ*_3_ are all less than 0, we can obtain

(18) $$\begin{array}{c} \left\{\begin{array}{c} C_{4}>B_{1},\\ C_{1}+G_{1}>P_{2},\\ \left(S_{2}-C_{2}\right)-\left(S_{1}-C_{3}\right)>D. \end{array}\right. \end{array}$$

The three inequalities in Equation (18) indicate that: (1) the cost of the general contractor's effort is greater than the owner's reward for its effort. (2) The sum of the cost of the government's active promotion and the subsidy to the owner is greater than the penalty for lack of effort on the part of the general contractor, meaning that government tends to choose a passive promotion strategy. (3) The net benefit of ICT adoption by the owner outweighs the value of impairment from passive promotion by the government.

(4) Stability analysis of (0,0,1) point

When (0,0,1) is ESS, the strategy chosen by the three parties is (passive promotion, passive adoption, effort), at this time *λ*_1_, *λ*_2_, *λ*_3_ are less than 0, we can obtain

(19) $$\begin{array}{c} \left\{\begin{array}{c} C_{4}<B_{1},\\ C_{1}+G_{2}-P_{1}>L,\\ D>\left(S_{2}-C_{2}\right)-\left(S_{1}-C_{3}\right). \end{array}\right. \end{array}$$

The three inequalities in Equation (19) indicate that: (1) the cost of the general contractor's effort is less than the owner's reward for its effort. (2) The total cost of active promotion by the government outweighs the social damage caused by choosing the passive strategy. (3) The net benefit to owners of ICT adoption is less than the value of the impairment to them from passive promotion by the government.

(5) Stability analysis of (1,1,0) point

When (1,1,0) is ESS and the strategy chosen by the three parties is (active promotion, proactive adoption, lack of effort), at which point *λ*_1_, *λ*_2_, *λ*_3_ are all less than 0, we can obtain

(20) $$\begin{array}{c} \left\{\begin{array}{c} C_{1}+G_{1}<P_{2},\\ C_{4}>B_{1}+G_{2}+P_{2},\\ S_{1}-\left(C_{3}+P_{1}\right)<S_{2}-C_{2}+G_{1}. \end{array}\right. \end{array}$$

The three inequalities in Equation (20) indicate that: (1) the sum of the cost of active promotion by the government and the subsidy to owners is less than the penalty for lack of effort by general contractors. (2) The cost of the general contractor's effort is greater than the sum of the owner's reward to it and the government subsidy. (3) The net benefit of owners choosing proactive adoption is greater than the net benefit of passive adoption.

(6) Stability analysis of (1,0,1) point

When (1,0,1) is ESS and the strategy chosen by the three parties is (active promotion, passive adoption, effort), at which point *λ*_1_, *λ*_2_, *λ*_3_ are all less than 0, we can obtain

(21) $$\begin{array}{c} \left\{\begin{array}{c} C_{1}+G_{2}-P_{1}<L,\\ C_{4}<B_{1}+G_{2}+P_{2},\\ S_{1}-\left(C_{3}+P_{1}\right)>S_{2}-C_{2}+G_{1}. \end{array}\right. \end{array}$$

The three inequalities in Equation (21) indicate that: (1) the total cost of active promotion by the government is less than the social damage caused by choosing the passive strategy. (2) The cost of general contractor's effort is less than the sum of the owner's reward to it and the government subsidy. (3) For owners, the net benefit of choosing passive adoption is greater than the net benefit of proactive adoption.

(7) Stability analysis of (0,1,1) point

When (0,1,1) is ESS and the strategy chosen by the three parties is (passive promotion, proactive adoption, effort), at which point *λ*_1_, *λ*_2_, *λ*_3_ are all less than 0, we can obtain

(22) $$\begin{array}{c} \left\{\begin{array}{c} C_{4}<B_{1},\\ D<\left(S_{2}-C_{2}\right)-\left(S_{1}-C_{3}\right)-C_{1}-G_{1}-G_{2}<0. \end{array}\right. \end{array}$$

The three inequalities in Equation (22) indicate that: (1) the cost of the general contractor's effort is less than the owner's reward for its effort. (2) The net benefit of ICT adoption by owners outweighs the value of impairment from passive promotion by the government. Therefore, owners choose the strategy of proactive adoption and general contractors choose the strategy of effort.

## 4. Numerical Simulation

In order to visualize the dynamic strategy evolution of the three stakeholders in the ICT adoption process, this paper uses MATLAB to conduct numerical simulations. Due to space limitations, we do not analyse all ESSs mentioned in Section 3.4. Based on industry life cycle theory [36], the life cycle of ICT adoption is divided into initial, development, and maturity stages.  Section 4.1 simulates and analyses the dynamic evolutionary rules of ICT adoption at different stages by varying the parameter settings to form different stability conditions. In Section 4.2, we discuss the impact of changes in the parameters of the evolutionary game model on the choice of strategies for the main subjects.

### 4.1. Results of Multistage Dynamic Evolution

In order to describe the dynamic evolution of the three stakeholder strategies at different stages of ICT adoption in China, we represented the evolutionary path of the three stages through numerical simulations. With reference to other relevant studies and satisfying different ESS establishment conditions, the parameters for each stage are set as shown in Table 5.

#### 4.1.1. Initial stage

**Figure 2:**
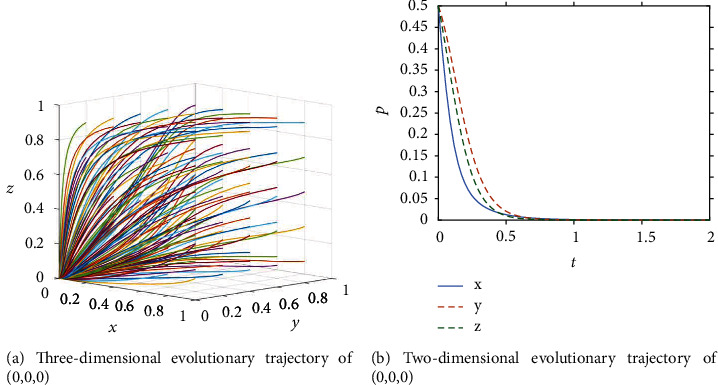
Evolution process of the system in the initial stage.

In the initial stages, the concept of ICT was initially promoted and publicized, with market research leading to technology development around specific directions such as BIM and digital design, smart sites, unmanned construction systems, and engineering big data platforms, but the government has yet to realize the importance of ICT adoption by stakeholders in the construction industry. At the same time, as ICT development is still in its nascent stage, the high costs and R&D expenses associated with the adoption of new technologies lead owners and general contractors to refuse to adopt ICT. Therefore, at this stage, the government chooses passive promotion, owners choose passive adoption, and general contractors choose the lack of effort strategy (ESS (0,0,0)). The analysis in Section 3.4 shows that the following three inequalities need to be satisfied for the point to become an ESS: *B*_1_ < *C*_4_, *L* + *P*_1_ + *P*_2_ < *C*_1_,*S*_2_ − *S*_1_ < *C*_2_ − *C*_3_ + *D*. Fifty sets of initial strategy combinations were randomly produced by MATLAB, and the paths of different initial strategy combinations evolving over time are shown in Figure 2(a). It can be seen that they all converge to S_1_(0,0,0) after iteration, which can prove that the ESS of government, owners and general contractors at this stage is (passive promotion, passive adoption, and lack of effort). Assuming that the initial probability of all three parties is 0.5, the evolutionary trajectory is shown in Figure 2(b), and the three reach the equilibrium state at a comparable rate, when *t* = 1, the three stakeholders reach the equilibrium state together along the evolutionary path.

#### 4.1.2. Development stage

**Figure 3:**
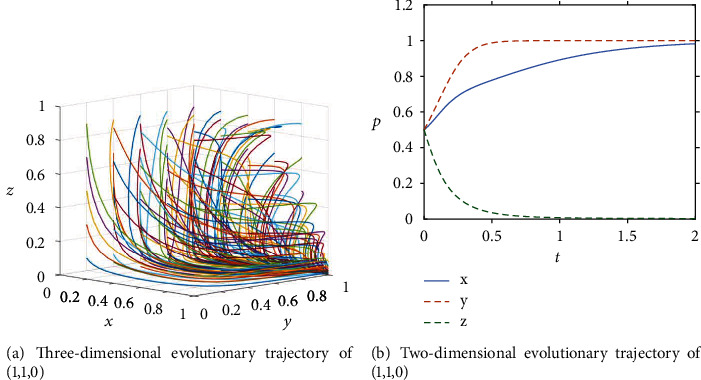
Evolution process of the system in the development stage.

In the mid-term of ICT adoption, relevant policies and regulations are gradually improved and ICT is deeply developed. From the application of BIM in the design phase to the application of IoT, 3D printing, and AI in the construction phase, as well as the application of cloud computing and big data in the operation and maintenance phase, the cost of ICT adoption gradually decreases as the technology continues to mature. Furthermore, as the government increases its policy bias and incentives, owners begin to realize the benefits of ICT adoption and will choose to proactively adopt ICT due to the high subsidies provided by the government and the penalties imposed on them for passive adoption. However, at the same time, the cost of the general contractor's effort is greater than the sum of the owner's rewards and government subsidies to them. Despite the increased penalties imposed by the government, general contractors continue to adopt a lack of effort strategy. At this point, the government chooses to actively promote, owners choose to adopt proactively, and general contractors choose the lack of effort strategy (ESS (1,1,0)). The conditions for the point to become ESS at this stage are:*C*_1_ + *G*_1_ < *P*_2_,*C*_4_ > *B*_1_ + *G*_2_ + *P*_2_,*S*_1_ − (*C*_3_ + *P*_1_) < *S*_2_ − *C*_2_ + *G*_1_. The paths of different initial strategy combinations evolving over time are shown in Figure 3(a). It can be seen that they all converge to S1(1,1,0) after iteration, proving that the ESS of the government, owner and general contractor become (active promotion, active adoption, lack of effort). Assuming that the initial probability of all three parties is 0.5, the evolutionary trajectory is shown in Figure 3(b), with the owner and the general contractor reaching equilibrium at almost the same rate and the government reaching equilibrium at a slower rate. When *t* = 2, the three stakeholders reach equilibrium together along the evolutionary path.

#### 4.1.3. Maturity stage

**Figure 4:**
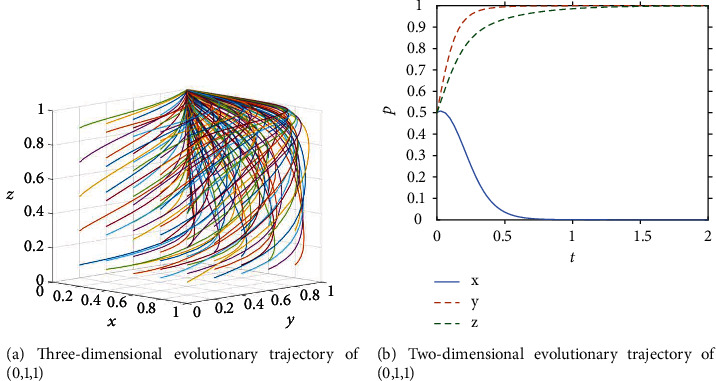
Evolution process of the system in the maturity stage.

As ICT continues to develop, a technology system for the entire industry chain has gradually been formed in the field of engineering and construction, comprising five stages: decision-making, design, production, construction, and operation and maintenance. The construction industry is thus equipped with intelligent self-awareness, self-learning, and self-determination, and is able to carry out continuous iterative optimization based on data and technology. In the maturity stage, where sound policies and regulations are in place and the cost of adopting new technologies is decreasing, ICT adoption develops in a virtuous cycle, with the benefits of ICT adoption far outweighing the costs to owners, and general contractors being rewarded for their efforts by owners and the excess profits generated by ICT adoption. At this point, the government's objective of promoting ICT adoption by owners and general contractors has largely been achieved, and the government begins to gradually reduce its financial investment in promoting ICT adoption and only advertises and encourages it. The government begins to tend to choose the strategy of passive promotion, owners choose the strategy of proactive adoption, and general contractors choose the strategy of effort ESS (0,1,1). At this stage, the conditions for the point to become ESS are: *C*_4_ < *B*_1_,*D* < (*S*_2_ − *C*_2_) − (*S*_1_ − *C*_3_).The paths of different initial strategy combinations evolving over time are shown in Figure 4(a). It can be seen that they all converge to S1(0,1,1) after iteration, which can prove that the ESS of the government, owner and general contractor become (passive promotion, active adoption, effort). Assuming that the initial probability of all three parties is 0.5, the evolutionary trajectory is shown in Figure 4(b), with the owner reaching equilibrium at a very fast rate, the government reaching equilibrium at a faster rate, and the general contractor reaching equilibrium at a slower rate as time continues to pass. When t =1.8, the three stakeholders reach equilibrium together along the evolutionary path.

### 4.2. Sensitivity Analysis of Model Parameters

In the ICT adoption process, besides the strategy choice of each stakeholder that affects the system to reach the ESS, changes in relevant parameters also affect the system evolution process. To investigate how changes in the main parameters affect the strategic choices of the game subjects, this study conducts a sensitivity analysis by changing the values of the parameters (G_1_, G_2_, P_1_, P_2_, C_2_, and C4). Taking the developmental stage of ICT adoption as the main example, we set the initial strategies of the three parties to (0.2,0.2,0.2).

#### 4.2.1. Impact of Government Financial Subsidy(G)

**Figure 5:**
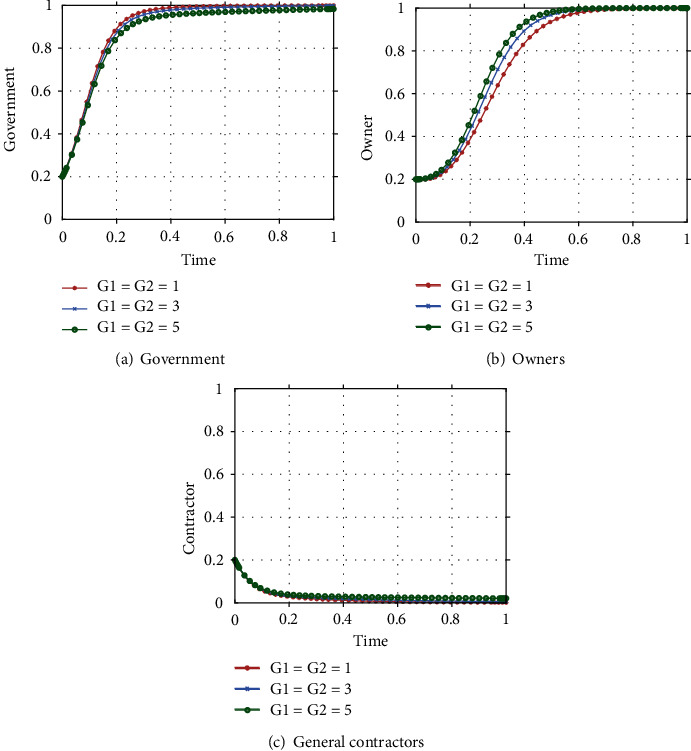
Impact of government financial subsidy G on the strategy evolution of the system.

Assuming that G_1_ = G_2_ = G is equal to 1, 3, 5, respectively, the evolutionary paths of the government, owner and general contractor strategy choices are shown in Figure 5 As the government's financial subsidies to owners and general contractors increase, the rate of government strategy evolution decreases to a certain extent, but eventually the government chooses to actively promote the adoption of ICT. During the evolutionary game, the government's strategic choices will be influenced by other factors, and as the social benefits of ICT adoption are still low at this stage, excessive financial subsidies will put greater financial pressure on the government, leading to a decrease in the rate at which the government reaches equilibrium. For owners, their strategy choice is positively influenced by the government's strategy choice and is more sensitive to financial subsidies, with the rate of evolution increasing as the amount of subsidies increases, but the degree of rate increase is not significant. It can be seen from Figure 5(c) that government subsidies at this stage do not influence the strategy choice of the general contractor. Therefore, a high level of government financial subsidies may not be the most desirable driving strategy at this stage of ICT development. The government should choose appropriate financial subsidies that can reduce the government's financial burden and at the same time drive owners to adopt ICT proactively.

#### 4.2.2. The impact of government penalty (P)

**Figure 6:**
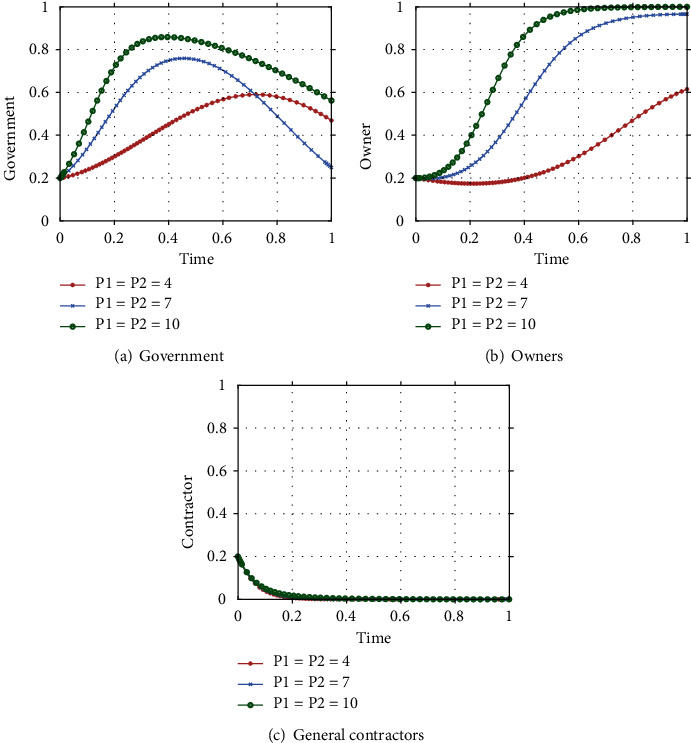
Impact of government penalty *P* on the strategy evolution of the system.

Assuming  *P*_1_ = *P*_2_ = *P* equal to 4, 7, 10, respectively, the evolutionary paths of the government, owner and general contractor strategy choices, with other parameters held constant, are shown in Figure 6. As shown in Figure 6(a), the increasing penalties increase the government's revenue, and to maintain that revenue, the government slows down its evolution towards equilibrium, showing a constant fluctuation.  Figure 6(b) reflects that the owner is highly influenced by government penalties, which reduce the owner's income and directly affect the owner's strategy choice. Under the government's active promotion strategy, the owner's strategy evolution rate keeps increasing in order to reduce costs and increase revenue as soon as possible. From Figure 6(c), it can be seen that the intensity of the government's penalty has little impact on the strategy evolution process of general contractors, and only when *P* = 10, the high penalty causes a slight decrease in the strategy evolution rate of general contractors, but they still choose the strategy of lack of effort. Therefore, in the process of promoting ICT adoption, the government should set reasonable penalties to protect its own revenue while acting as an incentive for owners and general contractors to take the initiative in ICT adoption.

#### 4.2.3. The impact of cost parameters (C_2_, C_4_)

**Figure 7:**
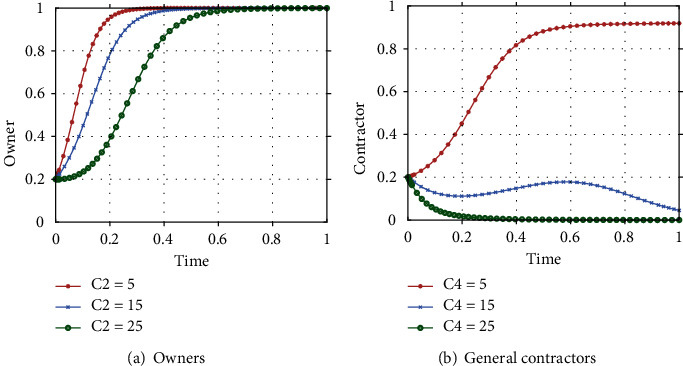
Impact of cost C_2_, C_4_ on the strategy evolution of the system.

In this research, the cost of ICT adoption by owners and general contractors is one of the main barriers driving technology adoption. Assuming C_2_ and C_4_ are taken to be 5, 15, and 25, respectively, the evolutionary path of the owner's and the general contractor's strategy choice is shown in Figure 7, with other parameters held constant. As can be seen from Figure 7(a), for owners, as the adoption cost decreases, the owner's strategy evolves at a faster rate and eventually reaches an equilibrium state of active adoption. As shown in Figure 7(b), the general contractor's sensitivity to ICT adoption costs is higher than that of the owner, and when C_4_ = 25, the general contractor chooses the strategy of lack of effort; when C_4_ = 15, the general contractor's strategy choice fluctuates continuously, but still tends to choose the strategy of lack of effort; as ICT continues to develop and mature, the adoption costs continue to decrease, and when C_4_ decreases to 5, the general contractor's willingness to choose the strategy of an effort increases significantly and reaches equilibrium as time advances.

## 5. Discussion

Through the analysis of the dynamic evolution of the three stages of ICT adoption in section 4.1, the ICT adoption strategy should be dynamically adjusted to the different stages. In the initial stage, the government is not yet aware of the importance of ICT, the research and development of new technologies is still in the exploration stage, and the high costs and R&D expenses associated with the adoption of new technologies lead owners and general contractors to refuse to adopt ICT. At this stage, it is necessary for the government to focus on cutting-edge developments in the industry and for relevant technology development companies to continue to increase investment and reduce adoption costs to enable ICT rollout. During the development phase, as the government increases its policy bias and incentives and the cost of ICT adoption gradually decreases, owners begin to adopt ICT on their own initiative, but the costs of adoption still outweigh the benefits for the general contractor. At this point, there is a need for greater government support for the general contractor, while the general contractor needs to recognise the benefits that ICT adoption can bring. In the maturity phase, technology development has entered a mature stage, the cost of adoption is decreasing, ICT adoption has formed a virtuous cycle with the government's upfront policy and financial support, owners and general contractors are aware of the benefits ICT brings and the construction industry is able to carry out continuous iterative optimization based on data and technology. In this context, the government begins to gradually reduce its financial investment in promoting ICT adoption and only advertises and encourages it.

According to current studies on technology adoption, the government must develop a policy combination of financial incentives, reputational rewards, and penalties to encourage technology use [29, 32], and the strength of subsidies that also can affect construction enterprises to adopt technology [28]. At the same time, the reduction in adoption costs can speed up the realization of ESS [32]. Based on the previous research results, it is the first in the field of promoting ICT adoption to establish a dynamic reward and penalty mechanism and promote technological innovation to reduce the cost of adoption.

1. Establish dynamic reward and penalty mechanisms

The promotion of ICT adoption requires not only sound policies and regulations and active cooperation from key stakeholders, but also government incentives and penalties play a key role. According to the results of 4.2.1 and 4.2.2, it can be seen that government financial subsidies for owners to proactively adopt ICT and penalties for passive adoption can effectively promote the adoption of ICT Larger penalties can significantly increase the willingness of owners to adopt ICT, while smaller penalties are not sufficient to promote the adoption of ICT. At the same time, financial subsidies act as a positive incentive for owners to adopt ICT, and appropriate subsidies can prevent owners from becoming dependent on the government and can also ease the government's financial burden. In addition, although the general contractor is less sensitive to financial subsidies and penalties, by increasing them is able to reduce the rate of evolution of the general contractor's strategy and increase the probability that it will choose to make an effort. At the same time, the owner's reward for the general contractor's effort can also incentivize its choice of effort. Therefore, the government can achieve an indirect incentive effect on the general contractor by dynamically adjusting the reward and penalty system for the owner. At different stages of ICT adoption, the government should make dynamic adjustments to the reward and penalty mechanism based on the results of the tripartite game model, in order to achieve optimal subsidy and penalty effects with limited fiscal expenditure, and to promote ICT development and implementation. Specifically, during the initial and developmental stages of ICT adoption, the government should provide appropriate financial subsidies and higher penalties to achieve ICT adoption. During the maturity stage, when ICT adoption has achieved a virtuous cycle and the benefits of ICT adoption for both owners and general contractors far exceed the costs, the government can gradually reduce the financial subsidies for both and choose an appropriate time to stop providing financial subsidies.

(2) Promoting science and technology innovation

Cost is one of the main factors influencing the choice of strategy for owners and general contractors. Currently, the low investment in R&D in the construction industry [3], which results in slow ICT development and high adoption costs. At a stage when ICT is not yet mature, the cost of adopting new technologies is high, and for rational participants, when the cost of adoption far outweighs the benefits, even if they will be penalized, it will not change their strategy of choosing passive adoption or making no effort. It can be found through this study that although owners are less sensitive to costs, by reducing costs, their strategy evolution rate keeps increasing in pursuit of profit maximization, which in turn achieves the aim of driving ICT adoption. It can be seen through Figure 7(b) that the general contractor is more sensitive to cost than the owner, which also provides some insight into the prioritization of driving ICT adoption. At the early stage of ICT development, priority should be given to research and development of intelligent construction technology, intelligent upgrading of production factors, technology and project management, development of intelligent equipment and intelligent machinery, and promotion of the project management mode of intelligent construction sites. In the middle and mature stages of ICT development, advanced information technologies such as IoT, big data, and AI should be vigorously promoted to form an integrated intelligent construction industry system by using ICT through standardized modelling, visualisation cognition and high-performance computing. For the government, the first step is to encourage high-tech enterprises to continuously research and innovate on ICT through economic means, such as technology subsidies and tax breaks. Secondly, it should build a technology research and development platform with the participation of multiple entities and encourage cooperation between schools and enterprises to promote the transformation of ICT, thereby reducing the cost of ICT adoption.

## 6. Conclusion

Exploring the dynamic behaviour of various stakeholders in the ICT adoption process is of great significance in accelerating the transformation and upgrading of China's construction industry. This research innovatively applies evolutionary game theory to the study of promoting ICT adoption by constructing a tripartite evolutionary game model among the government, owner and general contractor, analysing the stability of each subject's strategy choice, and using Lyapunov's stability theory to judge the stability of all possible equilibrium points. Finally, simulation analysis is used to verify the validity of the evolutionary game model and to study the influence of the main parameters of the model on the strategies of the three stakeholders.

We found that: (1) There are seven possible ESSs in the evolutionary game model, and the conditions for each ESS to hold mainly depend on the relationship between the costs and revenues of each stakeholder. (2) Based on the industry life cycle theory, the ICT adoption process is divided into the initial stage, development stage and maturity stage, and through analysis, it is found that three ESSs correspond to the three stages of ICT adoption, i.e. initial stage (ESS(0,0,0)), development stage (ESS(1,1,0)) and maturity stage (ESS(0,1,1)). (3) Owners are more sensitive to government subsidies and penalties than general contractors, and a dynamic reward and punishment mechanism should be established to achieve a win-win situation for all three parties. (4) General contractors are more sensitive to costs than owners, and the high costs associated with the adoption of new technologies will discourage general contractors from using ICT, so more attention should be paid to the incremental costs caused by ICT adoption.

This study examines the strategy evolution mechanism of various stakeholders at various stages of ICT adoption from a dynamic standpoint, which helps in understanding the relationship between stakeholders' behavioural strategies and ICT development and can promote ICT adoption in China's construction industry. The research results provide a theoretical basis for stakeholders to formulate and adjust their strategies in the ICT adoption process, and help the government to predict the behaviour of enterprises and improve the promotion mechanism. We believe that as ICT continues to evolve, other key stakeholders in the ICT technology adoption game, such as research institutions, may promote ICT implementation and adoption by strengthening collaboration between industry, academia and research. With the growing industrialisation of construction and the promotion of integrated building models, design units may also actively adopt ICT for building design. However, the specific behavioural strategies of these key stakeholders will require further research in the future. Therefore, this study still has the following shortcomings: (1) Only three stakeholders - government, owner and general contractor - have been considered, there may be other stakeholders in the ICT adoption process that require further analysis. (2) Only quantifiable and research-based parameters were considered in this paper for the model construction, and some objective social factors were not fully considered. (3) Future research can determine the parameter values through actual cases to make the model results more accurate.

## Figures and Tables

**Table 1:** The specific applications of Evolutionary Game Theory in three areas.

Research area	Author	Research content
Green building technology adoption	Yang et al. [28]	They developed an evolutionary game optimization model to reveal the game strategy changes of various stakeholders and the effects of environmental policies on the adoption of green building technologies (GBTs) among alliance-based construction enterprises in order to hasten the adoption of GBTs among construction enterprises.
	Chen et al. [29]	To clarify how policy influences GBT adoption, this study developed a subsidised evolutionary game model to determine how government policy with positive incentives influences GBT adoption, with government and construction stakeholders selected as game players.

BIM adoption	Liu [30]	Based on evolutionary game theory, Liu [30] analyses the evolutionary process between government and developers in BIM adoption decisions, with a view to validating the effectiveness of government-developer interaction in promoting BIM adoption through mathematical modelling.
	Jia et al. [31]	To promote the application of BIM technology in PPP projects, Jia et al. [31] established a tripartite evolutionary game model among the government, social capital, and contractors and analysed the key influencing factors of the equilibrium strategy through numerical simulation.

Prefabricated construction	Wang et al. [32]	To stimulate the adoption of prefabricated construction method, an evolutionary game model was established on the basis of variables influencing the strategy selection of game players for determining the effective incentive policies. The result shows that the incentives proposed by the government should not only target real estate enterprises but also focus on consumers, manufacturers of prefabricated components and contractors.
	Li et al. [33]	Li et al. [33] think that the main impediments of implementing prefabricated construction are initial cost and value justification of the whole project. Using evolutionary game theory and system dynamics, they develop an evolutionary game model to determine the revenue risk associated with the promotion of prefabricated construction.

**Table 2:** Description of major parameters.

Parameters	Meaning
*R*_1_	Social benefits to the government when only one of the owner and the general contractor proactively adopts ICT
*R*_2_	Social benefits to the government when both the owner and the general contractor proactively adopt ICT
*L*	Social damage caused by passive government promotion and passive adoption by owners
*C*_1_	The cost to the government when actively promoting
*G*_1_	Subsidies (land concessions, tax concessions, etc.) received by owners who proactively adopt when the government actively promotes ICT
*G*_2_	Subsidies (financial compensation, etc.) for the general contractor's efforts when the government actively promotes ICT
*P*_1_	Penalties for passive adoption of ICT by owners when actively promoted by the government
*P*_2_	Penalties for lack of effort by general contractors when actively promoted by the government
*D*	Impairment value for owners' proactive ICT adoption when government passively promotes it
*C*_2_	Costs paid by owners for proactive ICT adoption
*C*_3_	Excess cost to owners for passive ICT adoption
*C*_4_	Costs incurred by general contractor efforts
*S*_1_	The benefits of passive ICT adoption by owners
*S*_2_	Benefits from the owner's proactive adoption of ICT (including financial benefits, reputational benefits, etc.)
*S*_3_	Fixed income for general contractors
*B*_1_	Owner's reward for general contractor's efforts
*B*_2_	Value of impairment to the owner for ICT adoption due to lack of effort by general contractor

**Table 3:** Game payoff matrix.

Stakeholders		Owners	
		Proactive adoption (*y*)	Passive adoption (1 − *y*)
Government	General	*R*_2_ − *C*_1_ − *G*_1_ − *G*_2_,	*R*_1_ − *C*_1_ − *G*_2_ + *P*_1_,
	Contractors	*G*_1_ − *C*_2_ + *S*_2_ − *B*_1_,	*S*_1_ − *P*_1_ − *C*_3_ − *B*_1_,
	Effort (*z*)	*G*_2_ − *C*_4_ + *S*_3_ + *B*_1_	*G*_2_ − *C*_4_ + *S*_3_ + *B*_1_

Active promotion (*x*)	General	*R*_1_ − *C*_1_ − *G*_1_ + *P*_2_,	−*C*_1_ + *P*_1_ + *P*_2_,
	Contractors	*G*_1_ − *C*_2_ + *S*_2_ − *B*_2_,	*S*_1_ − *P*_1_ − *C*_3_ − *B*_2_,
	Lack of effort _(1-z)_	*S*_3_ − *P*_2_	*S*_3_ − *P*_2_

Government	General	*R*_2_,	*R*_1_ − *L*,
	Contractors	*S*_2_ − *C*_2_ − *B*_1_ − *D*,	*S*_1_ − *C*_3_ − *B*_1_,
	Effort (*z*)	*S*_3_ + *B*_1_ − *C*_4_	*S*_3_ + *B*_1_ − *C*_4_

Passive promotion (1 − *x*)	General	*R*_1_,	−*L*,
	Contractors	*S*_2_ − *C*_2_ − *B*_2_ − *D*,	*S*_1_ − *C*_3_ − *B*_2_,
	Lack of effort (1 − *z*)	*S*_3_	*S*_3_

**Table 4:** Eigenvalues and stability conditions for each equilibrium point.

Equilibrium points	Eigenvalue			Stability conditions	Results
	*λ*_1_	*λ*_2_	*λ*_3_		
(0,0,0)	B_1_-C_4_	L-C_1_ + P_1_ + P_2_	C_3_-C_2_-D-S_1_ + S_2_	*λ*_1、_ *λ*_2、_ *λ*_3_ < 0	ESS
(1,0,0)	C_1_-L-P_1_-P_2_	B_1_-C_4_ + G_2_ + P_2_	C_3_-C_2_ + G_1_ + P_1_-S_1_ + S_2_	*λ*_1、_ *λ*_2、_ *λ*_3_ < 0	ESS
(0,1,0)	B_1_-C_4_	P_2_-G_1_-C_1_	C_2_-C_3_ + D + S_1_-S_2_	*λ*_1、_ *λ*_2、_ *λ*_3_ < 0	ESS
(0,0,1)	C_4_-B_1_	L-G_2_-C_1_ + P_1_	C_3_-C_2_-D-S_1_ + S_2_	*λ*_1、_ *λ*_2、_ *λ*_3_ < 0	ESS
(1,1,0)	C_1_ + G_1_-P_2_	B_1_-C_4_ + G_2_ + P_2_	C_2_-C_3_-G_1_-P_1_ + S_1_-S_2_	*λ*_1、_ *λ*_2、_ *λ*_3_ < 0	ESS
(1,0,1)	C_1_ + G_2_-L-P_1_	C_4_-B_1_-G_2_-P_2_	C_3_-C_2_ + G_1_ + P_1_-S_1_ + S_2_	*λ*_1、_ *λ*_2、_ *λ*_3_ < 0	ESS
(0,1,1)	C_4_-B_1_	-C_1_-G_1_-G_2_	C_2_-C_3_ + D + S_1_-S_2_	*λ*_1、_ *λ*_2、_ *λ*_3_ < 0	ESS
(1,1,1)	C_1_ + G_1_ + G_2_	C_4_-B_1_-G_2_-P_2_	C_2_-C_3_-G_1_-P_1_ + S_1_-S_2_	*λ*_1、_ *λ*_2、_ *λ*_3_ < 0	Unstable

**Table 5:** The parameter values for each stage in the evolutionary game model.

Parameter	R_1_	R_2_	L	C_1_	G_1_	G_2_	P_1_	P_2_	D	C_2_	C_3_	C_4_	S_1_	S_2_	S_3_	B_1_	B_2_
Initial	5	10	6	15	5	5	2	2	4	25	10	15	25	35	20	5	10
Development	5	10	6	8	5	5	10	15	4	25	15	25	35	45	25	5	10
Maturity	5	10	6	0	5	5	4	4	4	10	6	5	35	50	25	8	4

## Data Availability

The data can be made available on a reasonable request by contacting the corresponding author.

## Supplementary Materials

Supplementary Materials The supplementary material is the MATLAB code for numerical simulation.
