# Supplementary material for: Intelligent Construction Technology Adoption Driving Strategy in China: A Tripartite Evolutionary Game Analysis
Source: J Environ Public Health. 2022 Oct 11;2022:9372443. doi: 10.1155/2022/9372443 (PMC9578914; doi:10.1155/2022/9372443)
Supplement: Supplementary Materials — The supplementary material is the MATLAB code for numerical simulation. [file 9372443.f1.pdf]

Figure 2(a):

```
figure(2);
R1=5,R2=10,L=6,C1=15,G1=5,G2=5,P1=2,P2=2,D=4,C2=25,C3=10,C4=15,S1=25,S2=35,S3=20
,B1=5,B2=10;
for i=0.1:0.2:1
    for j=0.1:0.2:1
        for k=0.1:0.2:1
            [t,y]=ode45(@(t,y)
sanfang(t,y,R1,R2,L,C1,G1,G2,P1,P2,D,C2,C3,C4,S1,S2,S3,B1,B2),[0,50],[i,j,k]);
            plot3(y(:,1),y(:,2),y(:,3),'linewidth',1);
            set(gca,'XTick',[0:0.2:1],'YTick',[0:0.2:1],'ZTick',[0:0.2:1])
            hold on
            axis([0 1 0 1 0 1])
            view([45 10])
        end
    end
end
grid on
hold on
xlabel('$x$','interpreter','latex');
ylabel('$y$','interpreter','latex');
zlabel('$z$','interpreter','latex','Rotation',360);
```

Figure 2(b):

```
x=0.5,y=0.5,z=0.5;
R1=5,R2=10,L=6,C1=15,G1=5,G2=5,P1=2,P2=2,D=4,C2=25,C3=10,C4=15,S1=25,S2=35,S3=20
,B1=5,B2=10;
[t,y]=ode45(@(t,y)
sanfang(t,y,R1,R2,L,C1,G1,G2,P1,P2,D,C2,C3,C4,S1,S2,S3,B1,B2),[0,2],[x,y,z]);
y1=y(:,1);
plot(t,y1,'-','color',[0.39216,0.58431,0.92941]);
hold on;
y2=y(:,2);
plot(t,y2,'-.','color',[0.82353,0.41176,0.11765]);
hold on;
y3=y(:,3);
plot(t,y3,'--','color',[0.13333,0.5451,0.13333]);
xlabel('t');
ylabel('p');
hold on;
legend('x','y','z');
```

Figure 3(a):

```
figure(3);
```

```
R1=5,R2=10,L=6,C1=8,G1=5,G2=5,P1=10,P2=15,D=4,C2=25,C3=15,C4=25,S1=35,S2=45,S3=2
5,B1=5,B2=10;
for i=0.1:0.2:1
    for j=0.1:0.2:1
        for k=0.1:0.2:1
            [t,y]=ode45(@(t,y)
sanfang(t,y,R1,R2,L,C1,G1,G2,P1,P2,D,C2,C3,C4,S1,S2,S3,B1,B2),[0,50],[i,j,k]);
            plot3(y(:,1),y(:,2),y(:,3),'linewidth',1);
            set(gca,'XTick',[0:0.2:1],'YTick',[0:0.2:1],'ZTick',[0:0.2:1])
            hold on
            axis([0 1 0 1 0 1])
            view([45 10])
        end
    end
end
grid on
hold on
xlabel('$x$','interpreter','latex');
ylabel('$y$','interpreter','latex');
zlabel('$z$','interpreter','latex','Rotation',360);
```

Figure 3(b):

```
x=0.5,y=0.5,z=0.5;
R1=5,R2=10,L=6,C1=8,G1=5,G2=5,P1=10,P2=15,D=4,C2=25,C3=15,C4=25,S1=35,S2=45,S3=2
5,B1=5,B2=10;
[t,y]=ode45(@(t,y)
sanfang(t,y,R1,R2,L,C1,G1,G2,P1,P2,D,C2,C3,C4,S1,S2,S3,B1,B2),[0,2],[x,y,z]);
y1=y(:,1);
plot(t,y1,'-','color',[0.39216,0.58431,0.92941]);
hold on;
y2=y(:,2);
plot(t,y2,'-.','color',[0.82353,0.41176,0.11765]);
hold on;
y3=y(:,3);
plot(t,y3,'--','color',[0.13333,0.5451,0.13333]);
xlabel('t');
ylabel('p');
hold on;
legend('x','y','z');
```

Figure 4(a):

```
figure(4);
R1=5,R2=10,L=6,C1=0,G1=5,G2=5,P1=4,P2=4,D=4,C2=10,C3=6,C4=5,S1=35,S2=50,S3=25,B1
=8,B2=4;
```

```
for i=0.1:0.2:1
        for j=0.1:0.2:1
                for k=0.1:0.2:1
                        [t,y]=ode45(@(t,y)
sanfang(t,y,R1,R2,L,C1,G1,G2,P1,P2,D,C2,C3,C4,S1,S2,S3,B1,B2),[0,50],[i,j,k]);
                        plot3(y(:,1),y(:,2),y(:,3),'linewidth',1);
                        set(gca,'XTick',[0:0.2:1],'YTick',[0:0.2:1],'ZTick',[0:0.2:1])
                        hold on
                        axis([0 1 0 1 0 1])
                        view([45 10])
                end
        end
end
grid on
hold on
xlabel('$x$','interpreter','latex');
ylabel('$y$','interpreter','latex');
zlabel('$z$','interpreter','latex','Rotation',360);
```

Figure 4(b):

```
x=0.5,y=0.5,z=0.5;
R1=5,R2=10,L=6,C1=0,G1=5,G2=5,P1=4,P2=4,D=4,C2=10,C3=6,C4=5,S1=35,S2=50,S3=25,B1
=8,B2=4;
[t,y]=ode45(@(t,y)
sanfang(t,y,R1,R2,L,C1,G1,G2,P1,P2,D,C2,C3,C4,S1,S2,S3,B1,B2),[0,2],[x,y,z]);
y1=y(:,1);
plot(t,y1,'-','color',[0.39216,0.58431,0.92941]);
hold on;
y2=y(:,2);
plot(t,y2,'-.','color',[0.82353,0.41176,0.11765]);
hold on;
y3=y(:,3);
plot(t,y3,'--','color',[0.13333,0.5451,0.13333]);
xlabel('t');
ylabel('p');
hold on;
legend('x','y','z');
```

Figure 5(government):

```
figure(5);
R1=5,R2=10,L=6,C1=8,G1=1,G2=1,P1=10,P2=15,D=4,C2=25,C3=15,C4=25,S1=35,S2=45,S3=2
5,B1=5,B2=10;
[t,y]=ode45(@(t,y)
sanfang(t,y,R1,R2,L,C1,G1,G2,P1,P2,D,C2,C3,C4,S1,S2,S3,B1,B2),[0,1],[0.2,0.2,0.2]);
```

```
points=1:1:length(t);
plot(t,y(:,1),'*-
','color',[0.80392,0.36078,0.36078],'linewidth',1,'markersize',5,'markerfacecolor','r','markerindices',
points);
hold on;
R1=5,R2=10,L=6,C1=8,G1=3,G2=3,P1=10,P2=15,D=4,C2=25,C3=15,C4=25,S1=35,S2=45,S3=2
5,B1=5,B2=10;
[t,y]=ode45(@(t,y)
sanfang(t,y,R1,R2,L,C1,G1,G2,P1,P2,D,C2,C3,C4,S1,S2,S3,B1,B2),[0,1],[0.2,0.2,0.2]);
points=1:1:length(t);
plot(t,y(:,1),'x-
','color',[0.39216,0.58431,0.92941],'linewidth',1,'markersize',5,'markerindices',points);
hold on;
R1=5,R2=10,L=6,C1=8,G1=5,G2=5,P1=10,P2=15,D=4,C2=25,C3=15,C4=25,S1=35,S2=45,S3=2
5,B1=5,B2=10;
[t,y]=ode45(@(t,y)
sanfang(t,y,R1,R2,L,C1,G1,G2,P1,P2,D,C2,C3,C4,S1,S2,S3,B1,B2),[0,1],[0.2,0.2,0.2]);
points=1:1:length(t);
plot(t,y(:,1),'o-
','color',[0.13333,0.5451,0.13333],'linewidth',1,'markersize',5,'markerindices',points);
hold on;
grid on
hold on
xlabel('$Time$','interpreter','latex','Rotation',0);
ylabel('$Government$','interpreter','latex');
set(gca,'XTick',[0:0.2:1],'YTick',[0:0.2:1]);
axis([0 1 0 1]);
legend('G1=G2=1','G1=G2=3','G1=G2=5');
```

Figure 5(owner):

```
figure(5);
R1=5,R2=10,L=6,C1=8,G1=1,G2=1,P1=10,P2=15,D=4,C2=25,C3=15,C4=25,S1=35,S2=45,S3=2
5,B1=5,B2=10;
[t,y]=ode45(@(t,y)
sanfang(t,y,R1,R2,L,C1,G1,G2,P1,P2,D,C2,C3,C4,S1,S2,S3,B1,B2),[0,1],[0.2,0.2,0.2]);
points=1:1:length(t);
plot(t,y(:,2),'*-
','color',[0.80392,0.36078,0.36078],'linewidth',1,'markersize',5,'markerfacecolor','r','markerindices',
points);
hold on;
R1=5,R2=10,L=6,C1=8,G1=3,G2=3,P1=10,P2=15,D=4,C2=25,C3=15,C4=25,S1=35,S2=45,S3=2
5,B1=5,B2=10;
[t,y]=ode45(@(t,y)
sanfang(t,y,R1,R2,L,C1,G1,G2,P1,P2,D,C2,C3,C4,S1,S2,S3,B1,B2),[0,1],[0.2,0.2,0.2]);
```

```
points=1:1:length(t);
plot(t,y(:,2),'x-
','color',[0.39216,0.58431,0.92941],'linewidth',1,'markersize',5,'markerindices',points);
hold on;
R1=5,R2=10,L=6,C1=8,G1=5,G2=5,P1=10,P2=15,D=4,C2=25,C3=15,C4=25,S1=35,S2=45,S3=2
5,B1=5,B2=10;
[t,y]=ode45(@(t,y)
sanfang(t,y,R1,R2,L,C1,G1,G2,P1,P2,D,C2,C3,C4,S1,S2,S3,B1,B2),[0,1],[0.2,0.2,0.2]);
points=1:1:length(t);
plot(t,y(:,2),'o-
','color',[0.13333,0.5451,0.13333],'linewidth',1,'markersize',5,'markerindices',points);
hold on;
grid on
hold on
xlabel('$Time$','interpreter','latex','Rotation',0);
ylabel('$Owner$','interpreter','latex');
set(gca,'XTick',[0:0.2:1],'YTick',[0:0.2:1]);
axis([0 1 0 1]);
legend('G1=G2=1','G1=G2=3','G1=G2=5');
```

Figure 5(contractor):

```
figure(5);
R1=5,R2=10,L=6,C1=8,G1=1,G2=1,P1=10,P2=15,D=4,C2=25,C3=15,C4=25,S1=35,S2=45,S3=2
5,B1=5,B2=10;
[t,y]=ode45(@(t,y)
sanfang(t,y,R1,R2,L,C1,G1,G2,P1,P2,D,C2,C3,C4,S1,S2,S3,B1,B2),[0,1],[0.2,0.2,0.2]);
points=1:1:length(t);
plot(t,y(:,3),'*-
','color',[0.80392,0.36078,0.36078],'linewidth',1,'markersize',5,'markerfacecolor','r','markerindices',
points);
hold on;
R1=5,R2=10,L=6,C1=8,G1=3,G2=3,P1=10,P2=15,D=4,C2=25,C3=15,C4=25,S1=35,S2=45,S3=2
5,B1=5,B2=10;
[t,y]=ode45(@(t,y)
sanfang(t,y,R1,R2,L,C1,G1,G2,P1,P2,D,C2,C3,C4,S1,S2,S3,B1,B2),[0,1],[0.2,0.2,0.2]);
points=1:1:length(t);
plot(t,y(:,3),'x-
','color',[0.39216,0.58431,0.92941],'linewidth',1,'markersize',5,'markerindices',points);
hold on;
R1=5,R2=10,L=6,C1=8,G1=5,G2=5,P1=10,P2=15,D=4,C2=25,C3=15,C4=25,S1=35,S2=45,S3=2
5,B1=5,B2=10;
[t,y]=ode45(@(t,y)
sanfang(t,y,R1,R2,L,C1,G1,G2,P1,P2,D,C2,C3,C4,S1,S2,S3,B1,B2),[0,1],[0.2,0.2,0.2]);
points=1:1:length(t);
```

```
plot(t,y(:,3),'o-
','color',[0.13333,0.5451,0.13333],'linewidth',1,'markersize',5,'markerindices',points);
hold on;
grid on
hold on
xlabel('$Time$','interpreter','latex','Rotation',0);
ylabel('$Contractor$','interpreter','latex');
set(gca,'XTick',[0:0.2:1],'YTick',[0:0.2:1]);
axis([0 1 0 1]);
legend('G1=G2=1','G1=G2=3','G1=G2=5');
```

Figure 6(government):

```
figure(6);
R1=5,R2=10,L=6,C1=8,G1=5,G2=5,P1=4,P2=4,D=4,C2=25,C3=15,C4=25,S1=35,S2=45,S3=25,
B1=5,B2=10;
[t,y]=ode45(@(t,y)
sanfang(t,y,R1,R2,L,C1,G1,G2,P1,P2,D,C2,C3,C4,S1,S2,S3,B1,B2),[0,1],[0.2,0.2,0.2]);
points=1:1:length(t);
plot(t,y(:,1),'*-
','color',[0.80392,0.36078,0.36078],'linewidth',1,'markersize',5,'markerfacecolor','r','markerindices',
points);
hold on;
R1=5,R2=10,L=6,C1=8,G1=5,G2=5,P1=7,P2=7,D=4,C2=25,C3=15,C4=25,S1=35,S2=45,S3=25,
B1=5,B2=10;
[t,y]=ode45(@(t,y)
sanfang(t,y,R1,R2,L,C1,G1,G2,P1,P2,D,C2,C3,C4,S1,S2,S3,B1,B2),[0,1],[0.2,0.2,0.2]);
points=1:1:length(t);
plot(t,y(:,1),'x-
','color',[0.39216,0.58431,0.92941],'linewidth',1,'markersize',5,'markerindices',points);
hold on;
R1=5,R2=10,L=6,C1=8,G1=5,G2=5,P1=10,P2=10,D=4,C2=25,C3=15,C4=25,S1=35,S2=45,S3=2
5,B1=5,B2=10;
[t,y]=ode45(@(t,y)
sanfang(t,y,R1,R2,L,C1,G1,G2,P1,P2,D,C2,C3,C4,S1,S2,S3,B1,B2),[0,1],[0.2,0.2,0.2]);
points=1:1:length(t);
plot(t,y(:,1),'o-
','color',[0.13333,0.5451,0.13333],'linewidth',1,'markersize',5,'markerindices',points);
hold on;
grid on
hold on
xlabel('$Time$','interpreter','latex','Rotation',0);
ylabel('$Government$','interpreter','latex');
set(gca,'XTick',[0:0.2:1],'YTick',[0:0.2:1]);
axis([0 1 0 1]);
```

```
legend('P1=P2=4','P1=P2=7','P1=P2=10');
```

Figure 6(owner):

```
figure(6);
R1=5,R2=10,L=6,C1=8,G1=5,G2=5,P1=4,P2=4,D=4,C2=25,C3=15,C4=25,S1=35,S2=45,S3=25,
B1=5,B2=10;
[t,y]=ode45(@(t,y)
sanfang(t,y,R1,R2,L,C1,G1,G2,P1,P2,D,C2,C3,C4,S1,S2,S3,B1,B2),[0,1],[0.2,0.2,0.2]);
points=1:1:length(t);
plot(t,y(:,2),'*-
','color',[0.80392,0.36078,0.36078],'linewidth',1,'markersize',5,'markerfacecolor','r','markerindices',
points);
hold on;
R1=5,R2=10,L=6,C1=8,G1=5,G2=5,P1=7,P2=7,D=4,C2=25,C3=15,C4=25,S1=35,S2=45,S3=25,
B1=5,B2=10;
[t,y]=ode45(@(t,y)
sanfang(t,y,R1,R2,L,C1,G1,G2,P1,P2,D,C2,C3,C4,S1,S2,S3,B1,B2),[0,1],[0.2,0.2,0.2]);
points=1:1:length(t);
plot(t,y(:,2),'x-
','color',[0.39216,0.58431,0.92941],'linewidth',1,'markersize',5,'markerindices',points);
hold on;
R1=5,R2=10,L=6,C1=8,G1=5,G2=5,P1=10,P2=10,D=4,C2=25,C3=15,C4=25,S1=35,S2=45,S3=2
5,B1=5,B2=10;
[t,y]=ode45(@(t,y)
sanfang(t,y,R1,R2,L,C1,G1,G2,P1,P2,D,C2,C3,C4,S1,S2,S3,B1,B2),[0,1],[0.2,0.2,0.2]);
points=1:1:length(t);
plot(t,y(:,2),'o-
','color',[0.13333,0.5451,0.13333],'linewidth',1,'markersize',5,'markerindices',points);
hold on;
grid on
hold on
xlabel('$Time$','interpreter','latex','Rotation',0);
ylabel('$Owner$','interpreter','latex');
set(gca,'XTick',[0:0.2:1],'YTick',[0:0.2:1]);
axis([0 1 0 1]);
legend('P1=P2=4','P1=P2=7','P1=P2=10');
```

Figure 6(contractor):

```
figure(6);
R1=5,R2=10,L=6,C1=8,G1=5,G2=5,P1=4,P2=4,D=4,C2=25,C3=15,C4=25,S1=35,S2=45,S3=25,
B1=5,B2=10;
[t,y]=ode45(@(t,y)
sanfang(t,y,R1,R2,L,C1,G1,G2,P1,P2,D,C2,C3,C4,S1,S2,S3,B1,B2),[0,1],[0.2,0.2,0.2]);
points=1:1:length(t);
```

```
plot(t,y(:,3),'*-
','color',[0.80392,0.36078,0.36078],'linewidth',1,'markersize',5,'markerfacecolor','r','markerindices',
points);
hold on;
R1=5,R2=10,L=6,C1=8,G1=5,G2=5,P1=7,P2=7,D=4,C2=25,C3=15,C4=25,S1=35,S2=45,S3=25,
B1=5,B2=10;
[t,y]=ode45(@(t,y)
sanfang(t,y,R1,R2,L,C1,G1,G2,P1,P2,D,C2,C3,C4,S1,S2,S3,B1,B2),[0,1],[0.2,0.2,0.2]);
points=1:1:length(t);
plot(t,y(:,3),'x-
','color',[0.39216,0.58431,0.92941],'linewidth',1,'markersize',5,'markerindices',points);
hold on;
R1=5,R2=10,L=6,C1=8,G1=5,G2=5,P1=10,P2=10,D=4,C2=25,C3=15,C4=25,S1=35,S2=45,S3=2
5,B1=5,B2=10;
[t,y]=ode45(@(t,y)
sanfang(t,y,R1,R2,L,C1,G1,G2,P1,P2,D,C2,C3,C4,S1,S2,S3,B1,B2),[0,1],[0.2,0.2,0.2]);
points=1:1:length(t);
plot(t,y(:,3),'o-
','color',[0.13333,0.5451,0.13333],'linewidth',1,'markersize',5,'markerindices',points);
hold on;
grid on
hold on
xlabel('$Time$','interpreter','latex','Rotation',0);
ylabel('$Contractor$','interpreter','latex');
set(gca,'XTick',[0:0.2:1],'YTick',[0:0.2:1]);
axis([0 1 0 1]);
legend('P1=P2=4','P1=P2=7','P1=P2=10');
```

Figure 7(owner):

```
figure(7);
R1=5,R2=10,L=6,C1=8,G1=5,G2=5,P1=10,P2=10,D=4,C2=5,C3=15,C4=25,S1=35,S2=45,S3=25
,B1=5,B2=10;
[t,y]=ode45(@(t,y)
sanfang(t,y,R1,R2,L,C1,G1,G2,P1,P2,D,C2,C3,C4,S1,S2,S3,B1,B2),[0,1],[0.2,0.2,0.2]);
points=1:1:length(t);
plot(t,y(:,2),'*-
','color',[0.80392,0.36078,0.36078],'linewidth',1,'markersize',5,'markerfacecolor','r','markerindices',
points);
hold on;
R1=5,R2=10,L=6,C1=8,G1=5,G2=5,P1=10,P2=10,D=4,C2=15,C3=15,C4=25,S1=35,S2=45,S3=2
5,B1=5,B2=10;
[t,y]=ode45(@(t,y)
sanfang(t,y,R1,R2,L,C1,G1,G2,P1,P2,D,C2,C3,C4,S1,S2,S3,B1,B2),[0,1],[0.2,0.2,0.2]);
points=1:1:length(t);
```

```
plot(t,y(:,2),'x-
','color',[0.39216,0.58431,0.92941],'linewidth',1,'markersize',5,'markerindices',points);
hold on;
R1=5,R2=10,L=6,C1=8,G1=5,G2=5,P1=10,P2=10,D=4,C2=25,C3=15,C4=25,S1=35,S2=45,S3=2
5,B1=5,B2=10;
[t,y]=ode45(@(t,y)
sanfang(t,y,R1,R2,L,C1,G1,G2,P1,P2,D,C2,C3,C4,S1,S2,S3,B1,B2),[0,1],[0.2,0.2,0.2]);
points=1:1:length(t);
plot(t,y(:,2),'o-
','color',[0.13333,0.5451,0.13333],'linewidth',1,'markersize',5,'markerindices',points);
hold on;
grid on
hold on
xlabel('$Time$','interpreter','latex','Rotation',0);
ylabel('$Owner$','interpreter','latex');
set(gca,'XTick',[0:0.2:1],'YTick',[0:0.2:1]);
axis([0 1 0 1]);
legend('C2=5','C2=15','C2=25');
```

Figure 7(contractor):

```
figure(7);
R1=5,R2=10,L=6,C1=8,G1=5,G2=5,P1=10,P2=10,D=4,C2=25,C3=15,C4=5,S1=35,S2=45,S3=25
,B1=5,B2=10;
[t,y]=ode45(@(t,y)
sanfang(t,y,R1,R2,L,C1,G1,G2,P1,P2,D,C2,C3,C4,S1,S2,S3,B1,B2),[0,1],[0.2,0.2,0.2]);
points=1:1:length(t);
plot(t,y(:,3),'*-
','color',[0.80392,0.36078,0.36078],'linewidth',1,'markersize',5,'markerfacecolor','r','markerindices',
points);
hold on;
R1=5,R2=10,L=6,C1=8,G1=5,G2=5,P1=10,P2=10,D=4,C2=25,C3=15,C4=15,S1=35,S2=45,S3=2
5,B1=5,B2=10;
[t,y]=ode45(@(t,y)
sanfang(t,y,R1,R2,L,C1,G1,G2,P1,P2,D,C2,C3,C4,S1,S2,S3,B1,B2),[0,1],[0.2,0.2,0.2]);
points=1:1:length(t);
plot(t,y(:,3),'x-
','color',[0.39216,0.58431,0.92941],'linewidth',1,'markersize',5,'markerindices',points);
hold on;
R1=5,R2=10,L=6,C1=8,G1=5,G2=5,P1=10,P2=10,D=4,C2=25,C3=15,C4=25,S1=35,S2=45,S3=2
5,B1=5,B2=10;
[t,y]=ode45(@(t,y)
sanfang(t,y,R1,R2,L,C1,G1,G2,P1,P2,D,C2,C3,C4,S1,S2,S3,B1,B2),[0,1],[0.2,0.2,0.2]);
points=1:1:length(t);
plot(t,y(:,3),'o-
```

```
','color',[0.13333,0.5451,0.13333],'linewidth',1,'markersize',5,'markerindices',points);
hold on;
grid on
hold on
xlabel('$Time$','interpreter','latex','Rotation',0);
ylabel('$Contractor$','interpreter','latex');
set(gca,'XTick',[0:0.2:1],'YTick',[0:0.2:1]);
axis([0 1 0 1]);
legend('C4=5','C4=15','C4=25');
```
